# Different spatial structure of plant‐associated fungal communities above‐ and belowground

**DOI:** 10.1002/ece3.10065

**Published:** 2023-05-21

**Authors:** Maria Faticov, Ahmed Abdelfattah, Peter Hambäck, Tomas Roslin, Ayco J. M. Tack

**Affiliations:** ^1^ Department of Ecology, Environment and Plant Sciences Stockholm University Stockholm Sweden; ^2^ Département de biologie Université de Sherbrooke Sherbrooke Quebec Canada; ^3^ Leibniz Institute of Agricultural Engineering and Bio‐economy Potsdam Germany; ^4^ Department of Ecology Swedish University of Agricultural Sciences Uppsala Sweden

**Keywords:** community ecology, dispersal, metacommunity, microclimate, phenology, phyllosphere microorganisms, soil microorganisms, spatial ecology

## Abstract

The distribution and community assembly of above‐ and belowground microbial communities associated with individual plants remain poorly understood, despite its consequences for plant–microbe interactions and plant health. Depending on how microbial communities are structured, we can expect different effects of the microbial community on the health of individual plants and on ecosystem processes. Importantly, the relative role of different factors will likely differ with the scale examined. Here, we address the driving factors at a landscape level, where each individual unit (oak trees) is accessible to a joint species pool. This allowed to quantify the relative effect of environmental factors and dispersal on the distribution of two types of fungal communities: those associated with the leaves and those associated with the soil of *Quercus robur* trees in a landscape in southwestern Finland. Within each community type, we compared the role of microclimatic, phenological, and spatial variables, and across community types, we examined the degree of association between the respective communities. Most of the variation in the foliar fungal community was found within trees, whereas soil fungal community composition showed positive spatial autocorrelation up to 50 m. Microclimate, tree phenology, and tree spatial connectivity explained little variation in the foliar and soil fungal communities. Foliar and soil fungal communities differed strongly in community structure, with no significant concordance detected between them. We provide evidence that foliar and soil fungal communities assemble independent of each other and are structured by different ecological processes.

## Graphical abstract

The distribution and community assembly of above‐ and belowground microbial communities associated with individual plants remains poorly understood, despite its consequences for plant–microbe interactions and plant health. To examine the effect of environmental factors and dispersal on the distribution of fungal communities, we sampled leaves and soil of *Quercus robur* trees, as well as microclimatic, phenological, and spatial variables, in a landscape in southwestern Finland. We provide evidence that foliar and soil fungal communities assemble independent of each other and are structured by different ecological processes.

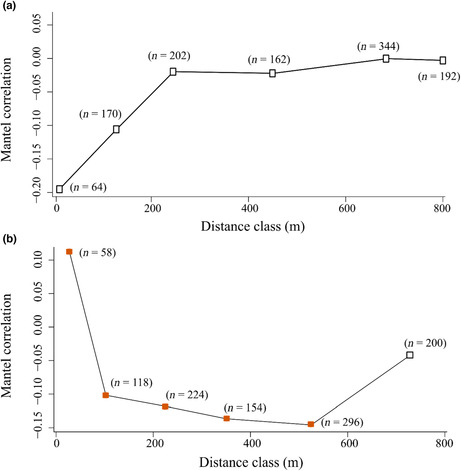

## 1 INTRODUCTION

Plants interact with a large diversity of microorganisms above‐ and belowground (Coince et al., 2014; Cordier et al., 2012; Jumpponen & Jones, 2010). These microbes play a major role in plant health and ecosystem processes like decomposition and carbon storage (Sterkenburg et al., 2018; Voříšková & Baldrian, 2013). Metabarcoding studies have demonstrated that microbial communities can be highly variable at the microscale, and show distinct biogeographical patterns at the global scale (Chua et al., 2018; Crowther et al., 2019; Thompson et al., 2017). Yet, such studies have mainly focused on either foliar or soil communities (but see, e.g., Bowman & Arnold, 2018, 2021; Coleman‐Derr et al., 2016). Understanding the spatial structure of, and a degree of association between, the foliar and soil microbial communities associated with single plant species is then a first step to understand the processes by which microbial communities assemble above‐ and belowground. As such, it is also a first advance towards predicting how a change in the state of relevant drivers will affect plant–microbe interactions, plant population dynamics, and ecosystem processes. Several environmental drivers, including climate, determine the structure of microbial communities both on the leaves and in the soil (Geml et al., 2016; Oita et al., 2021; Tedersoo et al., 2014; U'Ren et al., 2019; Zimmerman & Vitousek, 2012). It is therefore important to examine how these environmental factors may affect the distribution of above‐ and belowground microbial communities associated with plant populations.

Metabarcoding studies have consistently revealed high variation in foliar and soil microbial communities at the sub‐meter scale (Maciá‐Vicente & Popa, 2021; Mundra et al., 2015; Rasmussen et al., 2018; Saikkonen, 2007). Yet, clear biogeographical patterns in microbial communities have also been found at the regional, continental, and global scales (Millberg et al., 2015; Tedersoo et al., 2014). At the level of individual tree, studies have demonstrated that microbial communities could differ between leaves of the same plant individual. However, few studies have compared variation in the foliar community within and among plant individuals belonging to the same species. As one example, Cordier et al. (2012) demonstrated that 69% of variation in the foliar fungal community composition of the European beech (*Fagus sylvatica*) was found among leaves within a single tree. Likewise, Leff et al. (2015) showed that a large part of the variation in the foliar bacterial community composition was found within individual *Ginkgo biloba* trees. Such large variation in microbial communities within the tree canopy can be attributed to microclimatic variation within the canopy and to differences in leaf traits, age, and secondary chemistry (Arnold et al., 2000; Gripenberg & Roslin, 2005; Harrison et al., 2015; Rodriguez et al., 2009; Wagner et al., 2016). Clearly, when all or most of the variation is within plant individuals, we cannot expect a strong effect of individual‐level variation in the microbial community on plant health. Beyond the individual plant, we also lack the data that allow us to characterize the spatial structure of the microbial community associated with plants at the scale of plant populations and landscapes. A major question is thus how the foliar and soil microbial communities are distributed within and among plant individuals of the same species, and whether above‐ and belowground microbial communities show similar or distinct spatial structure.

Importantly, the relative role of different factors will likely differ with the scale examined (Lawler & Torgersen, 2020; Levin, 1992). At a landscape level, where all plant individuals are accessible to a joint species pool, variation in plant‐associated microbial communities can be related to metacommunity theory. Here, variation in microbial communities will likely reflect the relative effect of the local environment vs dispersal (Leibold et al., 2004; Ovaskainen et al., 2019). For example, the Baas Becking hypothesis states that microbes are strong dispersers and that local communities are mainly shaped by the filtering effect of local abiotic and biotic conditions (Becking, 1934; Cadotte & Tucker, 2017). Conceptually, this corresponds to one of the paradigms of metacommunity theory, the species sorting paradigm, where microbial species are sorted according to their environmental niche (Chase & Leibold, 2003). As one set of environmental factors, climatic variation at the landscape scale might explain spatial variation in microbial communities. Indeed, temperature, rainfall, and humidity have been shown to explain variation in foliar and soil communities at scales ranging from several hundred meters to thousands of kilometers (Coince et al., 2014; Millberg et al., 2015; Zimmerman & Vitousek, 2012). In accordance with this view, several studies have shown that environmental filtering affects soil microbial community composition more than dispersal limitation (Bahram et al., 2015; Cadotte & Tucker, 2017; Kivlin et al., 2014).

Despite this general view, the relative importance of environmental variables in shaping the spatial structure of microbial communities may vary among systems. Foliar and soil microbial communities may respond differently to environmental drivers and experience differences in dispersal limitation. As one example, Bowman and Arnold (2021) showed that the factors governing the distribution of foliar and soil fungi differed between functional guilds, where endophytic fungi were constrained by environmental factors, while ectomycorrhizal fungi were mainly limited by dispersal. Similarly, Junker et al. (2021) demonstrated that leaf bacterial communities were less dispersal limited than soil bacterial communities. This study also showed that high dispersal rates of leaf‐associated bacteria overrode the effects of spatial variation in environmental variables (Bowman & Arnold, 2018; Huang et al., 2016). Future studies might thus focus on differences in metacommunity processes between different microbial communities, where one hypothesis is that above‐ and belowground microbial communities are driven by different assembly processes. For example, due to high dispersal rates foliar microbial communities might conform to the mass effect paradigm (Choudoir et al., 2018), whereas soil communities adhere to a species‐sorting paradigm under which abiotic factors are dominant forces in structuring communities (Leibold & Wilbur, 1992).

Foliar and soil microbial communities are also linked through several mechanisms. First, leaf drop moves microorganisms from the canopy to the soil, and microorganisms can disperse (e.g., by wind or vectored by insects) from soil to leaves and from leaves to soil (Grady et al., 2019; Vorholt, 2012). Second, microorganisms in soil can thrive as endophytes and spread from the soil to the roots and then to the leaves through xylem vessels of the plant (Abdelfattah et al., 2021; Compant et al., 2016). Third, if foliar and soil microbial communities respond to the same environmental drivers and spatial processes, then there may be an association between spatial variation in the composition of one community and spatial variation in the other. Despite all these putative mechanisms, it is unclear whether links are strong enough to override the role of processes that might result in a weakening of the association between the above‐ and belowground microbial communities, such as environmental filtering caused by different growth conditions between leaves and soil, differences in the dispersal of spores originating from the air and soil and stochasticity.

To investigate differences in ecological processes structuring fungal communities associated with plant leaves and soil at the landscape scale, we sampled the leaves and soil of pedunculate oak (*Quercus robur*) trees on a 5 km^2^ island in southwestern Finland. Using these data, we tested the following hypotheses:

1. If soil fungal communities are more dispersal limited than foliar fungal communities, we would expect a stronger spatial structure in the former at the landscape scale. Despite the expectation for a weak spatial structure in the foliar fungal community at the landscape scale, we expected high within‐tree variation in the foliar fungal community due to high environmental heterogeneity among leaves.
2. To examine specific environmental drivers structuring foliar and soil microbial communities at the landscape scale, we focused on the role of microclimate (temperature and relative humidity), host characteristics (tree autumn phenology), and host distribution (spatial connectivity). We expected that variation in microclimate and host characteristics would explain the spatial structure of both foliar and soil fungal communities at the landscape scale but not necessarily in the same way. More specifically, we expected temperature and relative humidity to be dominant factors in structuring foliar fungal communities, and oak spatial connectivity in structuring soil fungal communities.
3. To examine the relative strength of processes that create links between leaves and soil on the same tree relative to other factors that structure microbial communities, we investigated the degree of association in spatial variation between these communities. We expected some degree of association between foliar and soil fungal communities.

## 2 MATERIALS AND METHODS

### 2.1 Study area

**FIGURE 1:**
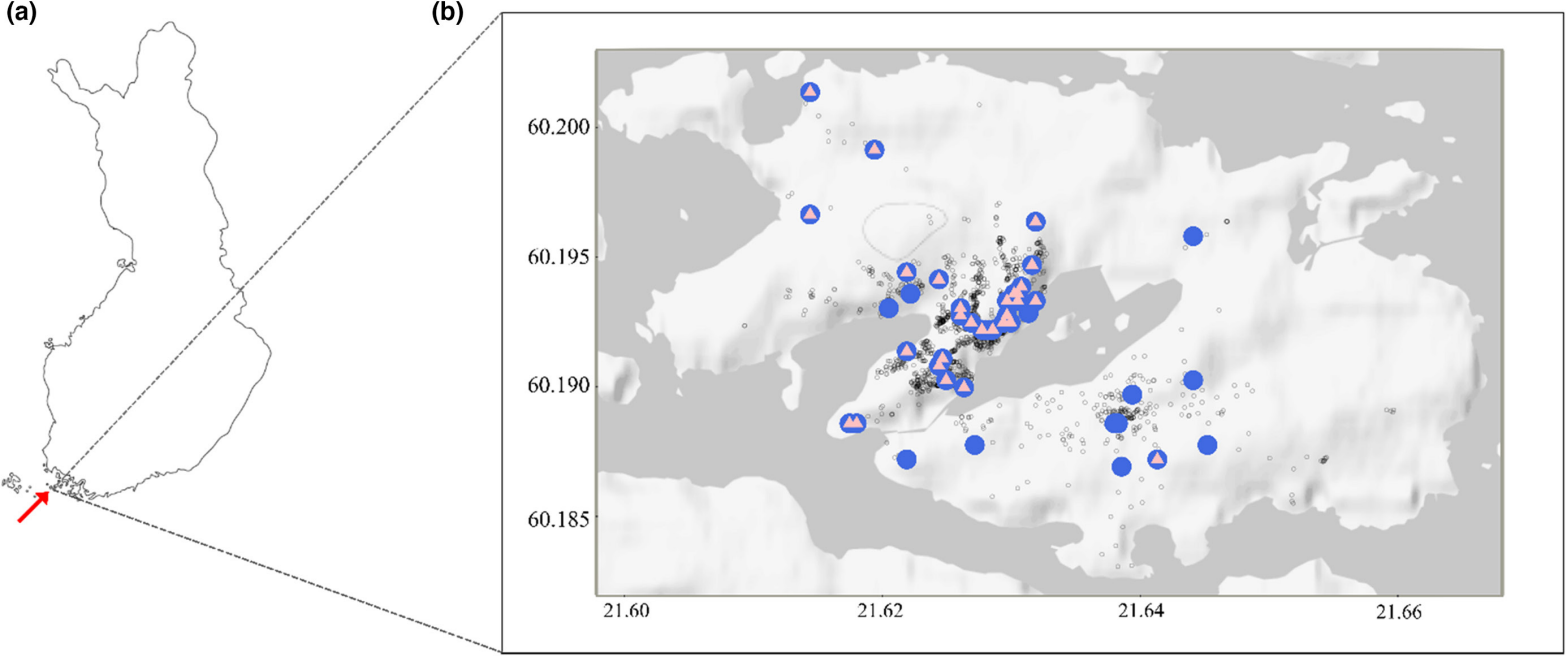
Location of sampled oak trees (*Quercus robur*) on the island Wattkast, southwestern Finland. (a) Map of Finland, with a red arrow pointing at the location of the island Wattkast in southwestern Finland. (b) Close‐up of the island of Wattkast, where blue circles represent the locations of the trees from which leaf and soil samples were collected. To assess the effect of local climatic conditions on foliar and soil fungal communities, we installed dataloggers on 27 oak trees (pink triangles). Small gray circles show the locations of all oak trees on the island (*n* = 1868).

We conducted our study on the 5 km^2^ island Wattkast in southwestern Finland (60°12′0′′N, 21°38′0′′E: Figure 1a), where the pedunculate oak *Q. robur* reaches its northern distribution limit. On this island, all 1868 oak trees higher than 0.5 m have previously been mapped (Gripenberg & Roslin, 2005). There are several dense oak stands, but also a high number of more isolated oak trees scattered throughout the landscape (Figure 1b). Other dominant trees on the island are silver birch (*Betula pendula*), Norway spruce (*Picea abies*), and Scots pine (*Pinus sylvestris*).

### 2.2 Sampling design

To assess within and among tree variation in the foliar fungal community, we randomly collected and individually stored 4 to 7 leaves from each of 19 trees. To investigate the spatial distribution and drivers of the foliar and soil fungal community, we also collected leaf and soil samples at the tree level for each of 49 trees (Figure 1b). We sampled the foliar fungal community by collecting and subsequently pooling 10 randomly selected leaves from each tree. For the soil fungal community, we took a soil core (c. 5‐cm diameter and 10‐ to 20‐cm deep, depending on the presence of rocks) at four randomly selected locations within the dripline of the tree canopy (i.e., below the branches), which were subsequently pooled and mixed. The leaf and soil samples were collected in September 2018. As tracing or identifying the roots of individual oak trees was infeasible in the rocky soil, the soil samples will represent a blend of microorganisms associated with the roots of oak and with other plant species living closely with the oak individual. We refer to them as “soil fungal communities.” Leaf samples were stored in Ziploc bags with silica gel, and soil samples were stored in the freezer until further molecular analysis.

To characterize variation in temperature and relative humidity in the tree canopy, we placed EL‐USB‐2 Lascar dataloggers (Lascar Electronics) on one to five low‐hanging branches on each of 27 oak trees (Figure 1b). To capture variation in soil temperature among trees, we installed iButtons (DS1921G; Maxim Integrated) at a depth of 5 cm below the canopy of 27 oak trees (Figure 1b). For the canopy and soil dataloggers, we calculated three biologically‐relevant bioclimatic variables by averaging temperature for the growing (May–September) and nongrowing season (November–March), as well as temperature seasonality (standard deviation of monthly mean temperature) for the year preceding data collection. For canopy dataloggers, which also recorded relative humidity, we further calculated growing and nongrowing season relative humidity. To estimate tree‐level variation in autumn phenology, we scored leaf discoloration (i.e., the proportion of the leaf that is brown) on 10 leaves on each of 19 trees in autumn 2018. Leaf discoloration was then averaged across the leaves of each tree. To characterize the spatial clustering of oak trees (e.g., whether trees grow in isolation or surrounded by many other oaks), we calculated the spatial connectivity for each tree by using the connectivity metric modified from Hanski (1999):

$$S_{i}=\sum_{i\neq j}N_{j}e^{-\alpha d_{\mathrm{ij}}},$$

where j ranges over all trees on the island (1868 trees; Gripenberg & Roslin, 2005), $N_{j}$ is the number of leaves on each tree, and $d_{\mathrm{ij}}$ is the distance between the focal tree i and tree j in meters. The parameter α was set to 1/100 m and $N_{j}$ was estimated using the formula log (number of leaves) = 0.92 + 2.55 × log (circumference at breast height in cm) (Gripenberg et al., 2008; Tack et al., 2010; Zheng et al., 2015).

### 2.3 Molecular methods

Leaf samples were grinded using a ball mill (Retsch Mixer Mill MM400), and 10 mg was used for DNA extraction using NucleoSpin Plant II kit (Machery‐Nagel) following the standard protocol. The soil samples were thoroughly homogenized and 25 mg was used for DNA extraction using DNeasy PowerSoil isolation kit (Qiagen). To characterize the foliar and soil fungal communities, we used primers targeting the internal transcribed spacer (ITS2) region (Schoch et al., 2012). We used the forward primer fITS7 (Ihrmark et al., 2012) and reverse primer ITS4 (White et al., 1990), which target a 250‐ to 450‐bp fragment encompassing the entire ITS2. For PCR amplification, PCR reactions were run in a volume of 25 μL and the reaction mixtures were prepared using Kapa HiFi Mastermix (Kapa Biosystems). The final product was sent to sequencing at SciLifeLab/NGI on Illumina MiSeq (Illumina Inc.) with 2 × 300 bp reads. For more details on the library preparation, see Supporting Information.

In total, we obtained 3,247,020 sequences from 192 samples after quality filtering and removal of sequences that appeared in negative controls. Three samples were removed due to mislabeling. The sequences were clustered into 12,956 amplicon sequence variants (ASVs) using DADA2 (Callahan et al., 2016). On average, fungal communities were represented by 16,911 reads per sample. The number of sequences varied among samples, ranging from 101 to 115,516 reads. The most abundant sequence in each ASV was identified using the UNITE database V8 released on February 2, 2019 (Abarenkov et al., 2010). When calculating fungal species richness (number of ASVs per sample) and Pielou's evenness (Pielou, 1966), we accounted for uneven sequencing depth by rarefying each sample to 300 reads, which allowed the inclusion of 185 samples. From these 185 samples, 101 samples were individual leaf samples, and 43 and 41 tree‐level leaf and soil samples, respectively. For community composition analyses, we used MetagenomeSeq's cumulative sum scaling as a normalization method to account for uneven sequencing depth (Paulson et al., 2013).

To classify ASVs into ecologically meaningful functional groups, we manually assigned functional guilds to 2296 ASVs, which made up 95% of the total number of reads. The assignment was done based on taxonomic identity or similarity of fungal species to the UNITE and NCBI database sequences recorded from well‐defined substrates (i.e., leaves and soil). For the fungal taxa found in leaves and soil, we used the following six functional groups: (i) yeasts, (ii) putative fungal pathogens (fungi that cause plant disease), (iii) putative saprotrophic or symbiotrophic fungi (fungi that break down organic matter or have a mutualistic relationship with plants), (iv) other fungi (e.g., fungi attacking plant pathogens), (v) fungi with unknown functions from the phylum Ascomycota and Basidiomycota, and (vi) unidentified fungi. For the soil fungi, we additionally used the functional group ectomycorrhizal fungi. We assigned yeasts to a separate guild because they can belong to several functional guilds (Canini et al., 2021; Kemler et al., 2017). For each sample, the relative abundance of each fungal guild was calculated as the ratio between the summed number of reads of all ASVs in a given guild and the total number of reads. A detailed description of the assigned functional guilds can be found in Table S1.

For full details on the molecular methods and bioinformatics, see Supporting Information. Sequencing data for each sample in this study are deposited at NCBI.

### 2.4 Statistical analyses

To investigate how the foliar fungal community varies within and among trees, we modeled species richness, evenness, and community composition as functions of “*Tree ID*.” For the models on species richness and evenness, we defined tree identity as a random effect. We used *lmer* in the package *lme4* (Bates et al., 2015). As random effects cannot be specified in *adonis2*, we defined tree identity as a fixed effect for community composition analysis. Variation among trees was characterized by dividing the variation attributed to tree identity by the total variation (tree‐level variation + residual variation). We thereby arrived at an intraclass correlation coefficient (Sokal & Rohlf, 1995), and within‐tree variation was calculated as one minus the intraclass correlation coefficient.

To quantify the percentage of variation in community composition that can be explained by spatial structure at the landscape scale, we used Moran's eigenvectors. This approach allowed us to simultaneously assess multiple spatial structures (Borcard & Legendre, 2002). We first generated a set of Moran's eigenvectors from the coordinates of each sampling point using distance‐based Moran's eigenvector maps (MEMs) (Legendre & Legendre, 2012). We identified positive MEMs that significantly (*p* < .05) described spatial patterns using the function *moran.randtest* in the *adespatial* package. To choose the MEMs that were important in structuring the fungal community, we performed forward selection independently for the foliar and soil data, using the approach proposed by Blanchet et al. (2008). To explore whether there is spatial autocorrelation in foliar and soil fungal species richness, evenness, and relative abundance of functional guilds, we used Moran's test implemented in the function *Moran. I* in the package *ape* (Paradis & Schliep, 2018). We visualized spatial autocorrelation in the foliar and soil fungal species richness, evenness, and relative abundance of functional guilds with the function *variogram* from the *gstat* package (Gräler et al., 2016; Pebesma, 2004). To examine spatial autocorrelation in foliar and soil fungal community composition, as well as in the composition of the subset of fungal guilds with a well‐defined functional role (i.e., yeasts, fungal pathogens, and ectomycorrhizal fungi), we used a Mantel test, as implemented in the function *mantel* in the *geosphere* package. To visualize spatial autocorrelation in the foliar and soil fungal community composition and in the composition of the subset of fungal guilds, we used Mantel correlograms as implemented in the function *mantel* in the *vegan* package.

To examine the effects of microclimate, autumn phenology, and host distribution on the fungal community in the leaves, we modeled fungal richness, evenness, community composition, and relative abundance of functional guilds as functions of growing season and nongrowing season temperature, growing and nongrowing season relative humidity, temperature seasonality, autumn phenology, and tree spatial connectivity. To identify how microclimate and tree connectivity affected the fungal community in the soil, we modeled fungal richness, evenness, community composition, and relative abundance of functional guilds as functions of growing and nongrowing season temperature, temperature seasonality, and tree spatial connectivity. For the univariate response variables such as species richness, evenness, and relative abundance of yeasts, fungal pathogens, and ectomycorrhizal fungi, we fitted linear models using the function *lm*. We used forward selection to arrive at the final models, where variables were added sequentially until all the variables to consider had *p* values below .05. Variance inflation factors were lower than the recommended cut‐off value of 3 in all final models, indicating that multicollinearity was not interfering strongly with model outcomes (all VIF < 3; Zuur et al., 2009). For the multivariate response variable community composition, we used the function *adonis2* in the *vegan* package (Oksanen et al., 2022; R Core Team, 2022). All models were run using Bray–Curtis dissimilarity metrics.

To investigate whether fungal species richness and evenness differed between leaves and soil, we used linear mixed models, where we modeled species richness and evenness as a function of “*Sample_type*” (e.g., leaves and soil). We accounted for variation among the different locations by including the random effect of “*Tree ID*” To test whether fungal community composition differed between leaves and soil, we used the function *adonis2* in the *vegan* package. To investigate the degree of association in spatial variation between fungal communities in the leaves and soil, we used a Procrustes analysis (Peres‐Neto & Jackson, 2001). First, we evaluated the degree of concordance between two matrices, that is, whether they represent similar information. Thereby we computed the Procrustes statistic, m^2^. Second, we used function *protest* from the *vegan* package to estimate the significance of the Procrustes statistic (Jackson, 1995; Oksanen et al., 2022). All analyses were conducted in R version 3.6.0 (R Core Team, 2022).

## 3 RESULTS

**FIGURE 2:**
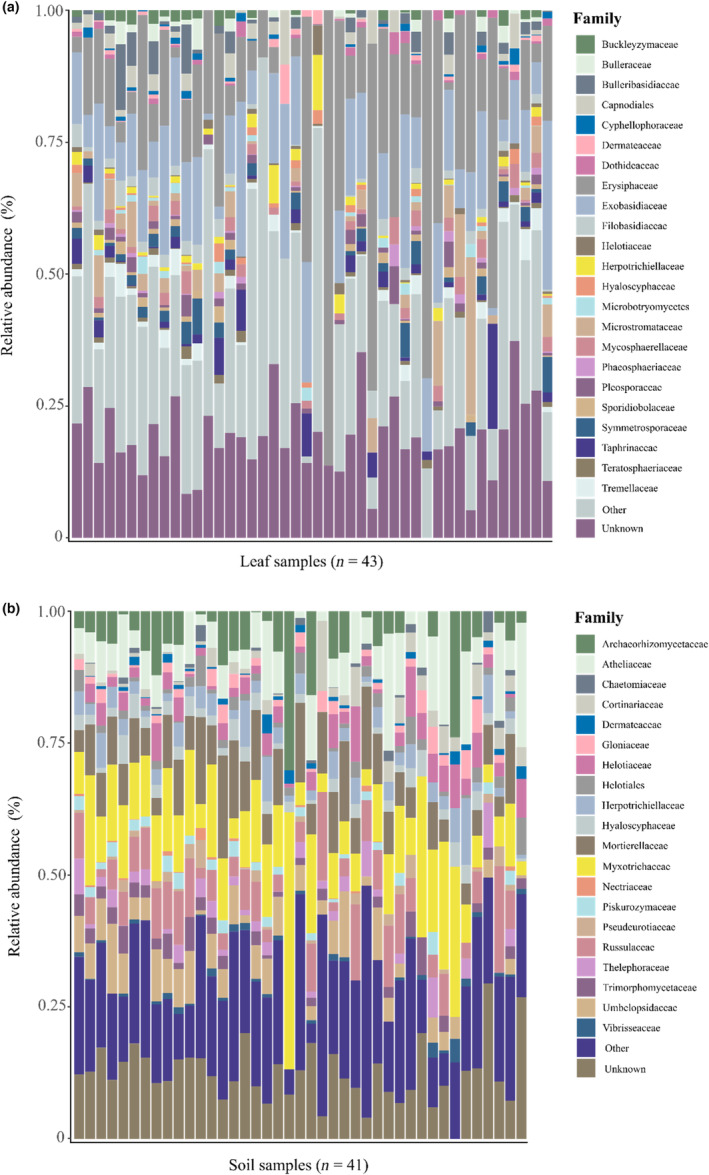
Stacked bar charts showing the relative abundance of fungal families in (a) leaves (*n* = 43 trees) and (b) soil (*n* = 41) of the pedunculate oak *Quercus robur*. Families with low relative abundance (<5%) were merged under the category “Other,” while the category “Unknown” represents taxa for which a putative taxonomic classification could not be assigned. Charts were generated using cumulative sum scaling‐normalized abundance matrices.

The phyla Ascomycota and Basidiomycota were dominant in both leaves and soil (Figure 2).

Among foliar fungi, Capnodiales, Cyphellophoraceae, Erysiphaceae, and Mycosphaerellaceae were the most abundant families in the phylum Ascomycota, whereas Bulleribasidiaceae, Exobasidiaceae, and Filobasidiaceae were the most abundant families in the phylum Basidiomycota (Figure 2a). Among soil fungi, Archaeorhizomycetaceae, Chaetomiaceae, Dermateaceae, and Gloniaceae were the most abundant families in the phylum Ascomycota, whereas Atheliaceae, Cortinariaceae, Russulaceae, and Thelephoraceae were the dominant families in the phylum Basidiomycota (Figure 2b).

### 3.1 Spatial patterns and drivers of foliar and soil fungal communities

**FIGURE 3:**
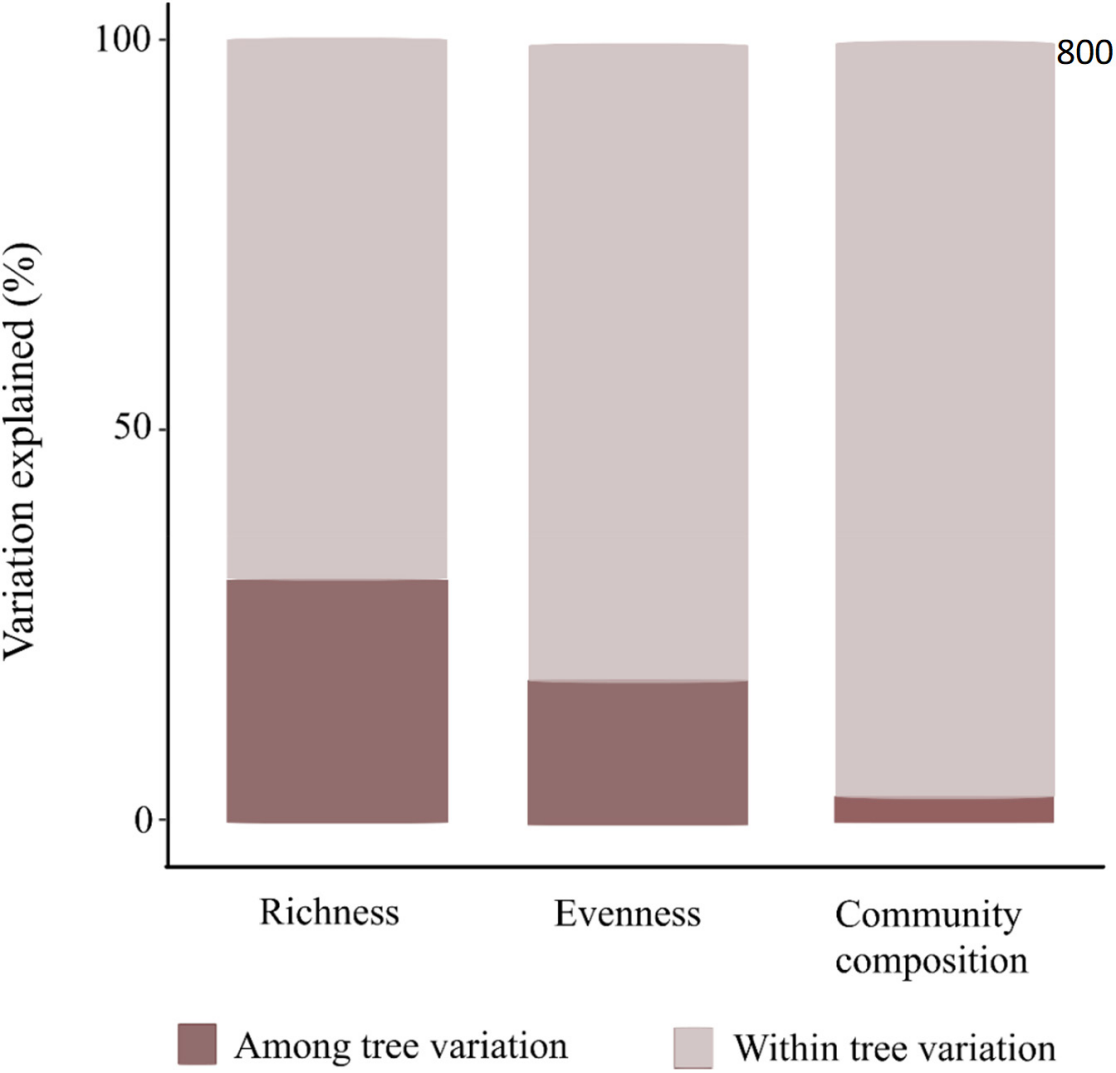
The percentage of variation in foliar fungal richness, evenness, and community composition found within and among trees of the pedunculate oak *Quercus robur*.

Foliar fungal communities were highly variable within trees, with 70% of the variation in species richness, 82% in evenness, and 98% in community composition found within trees (Figure 3).

**FIGURE 4:**
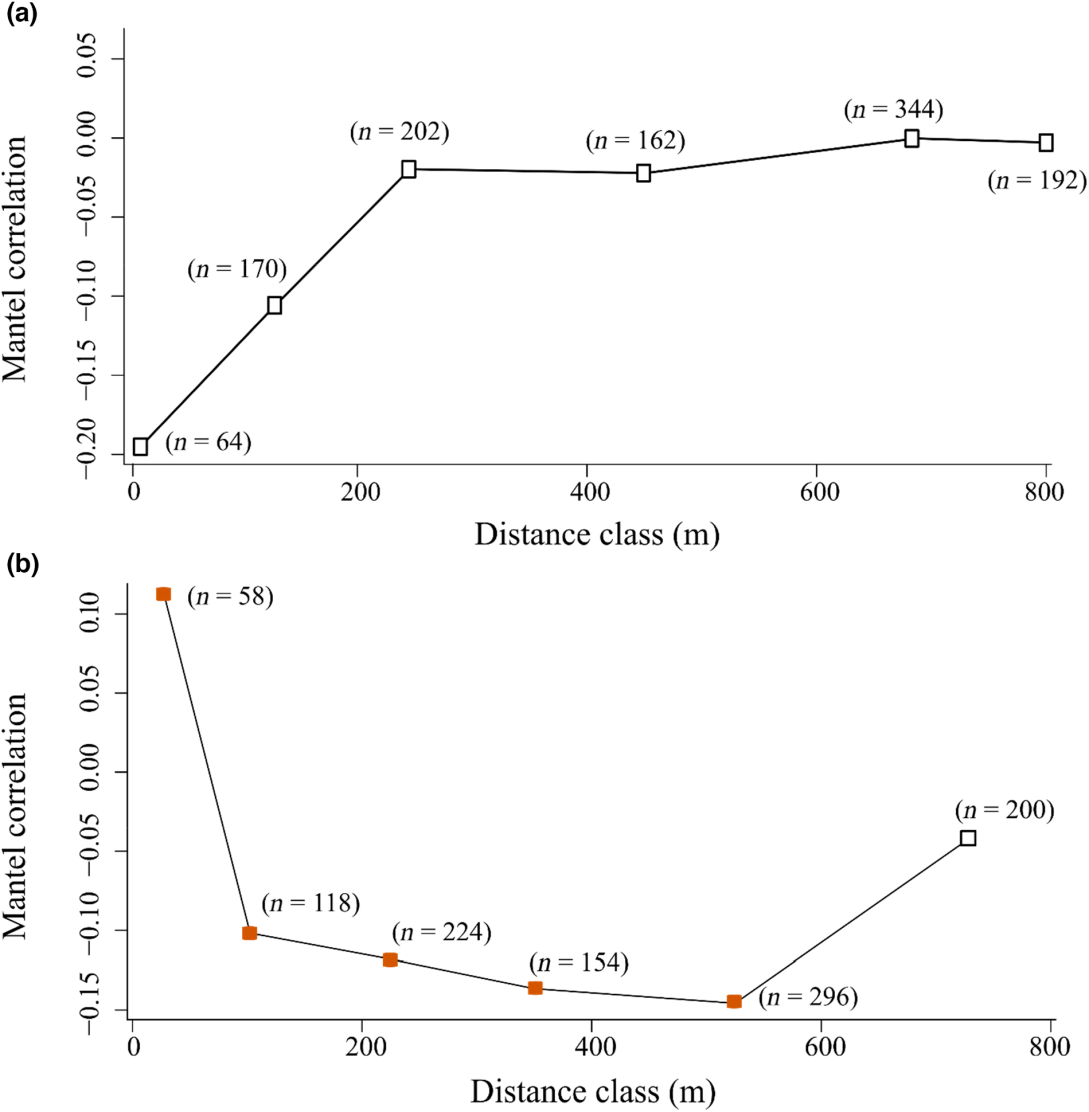
Mantel correlogram illustrating spatial autocorrelation of the (a) leaf and (b) soil fungal community composition among trees (*Quercus robur*) on the island Wattkast. Shown are Mantel correlation coefficients for each of six distance classes. Significant (*p* < .05) and nonsignificant values are indicated by dark orange and white squares, respectively. The number of pairwise distances within each class is given in brackets.

MEMs revealed a stronger spatial structure in the composition of fungal communities associated with soil than with foliage, with 5% and 11% of the variation in community composition explained by spatial variables, respectively. Consistent with these patterns, we found no spatial autocorrelation in the foliar fungal community composition (*r* = −0.01, *p* = .706, Figure 4a), whereas the soil fungal community composition showed positive spatial autocorrelation up to 50 m (*r* = 0.20, *p* = .02, Figure 4b). We found no spatial autocorrelation in the foliar and soil fungal richness (*p* = .46 and .91), evenness (*p* = .55 and .07), and the relative abundance and composition of the functional guilds in the leaves and soil (Table S4; Figures S1–S3).

None of the microclimatic predictors (i.e., growing and nongrowing season temperature, growing and nongrowing season relative humidity, and temperature seasonality) were related to fungal species richness, evenness, community composition or the relative abundance of functional guilds in the leaves, respectively (Tables S2 and S3; Figure S4). Similarly, the temperature in the growing and nongrowing season and temperature seasonality did not detectably affect fungal richness, evenness, community composition, and relative abundance of functional guilds in the soil (Tables S2 and S3; Figure S5). We did not detect any effect of autumn phenology and oak spatial connectivity on foliar fungal richness, evenness, community composition, and relative abundance of functional guilds (Tables S2 and S3; Figure S4). Neither did we detect any effect of oak spatial connectivity on soil fungal richness, evenness, community composition, and relative abundance of functional guilds (Tables S2 and S3; Figure S5).

### 3.2 Relationship between foliar and soil fungal communities

**FIGURE 5:**
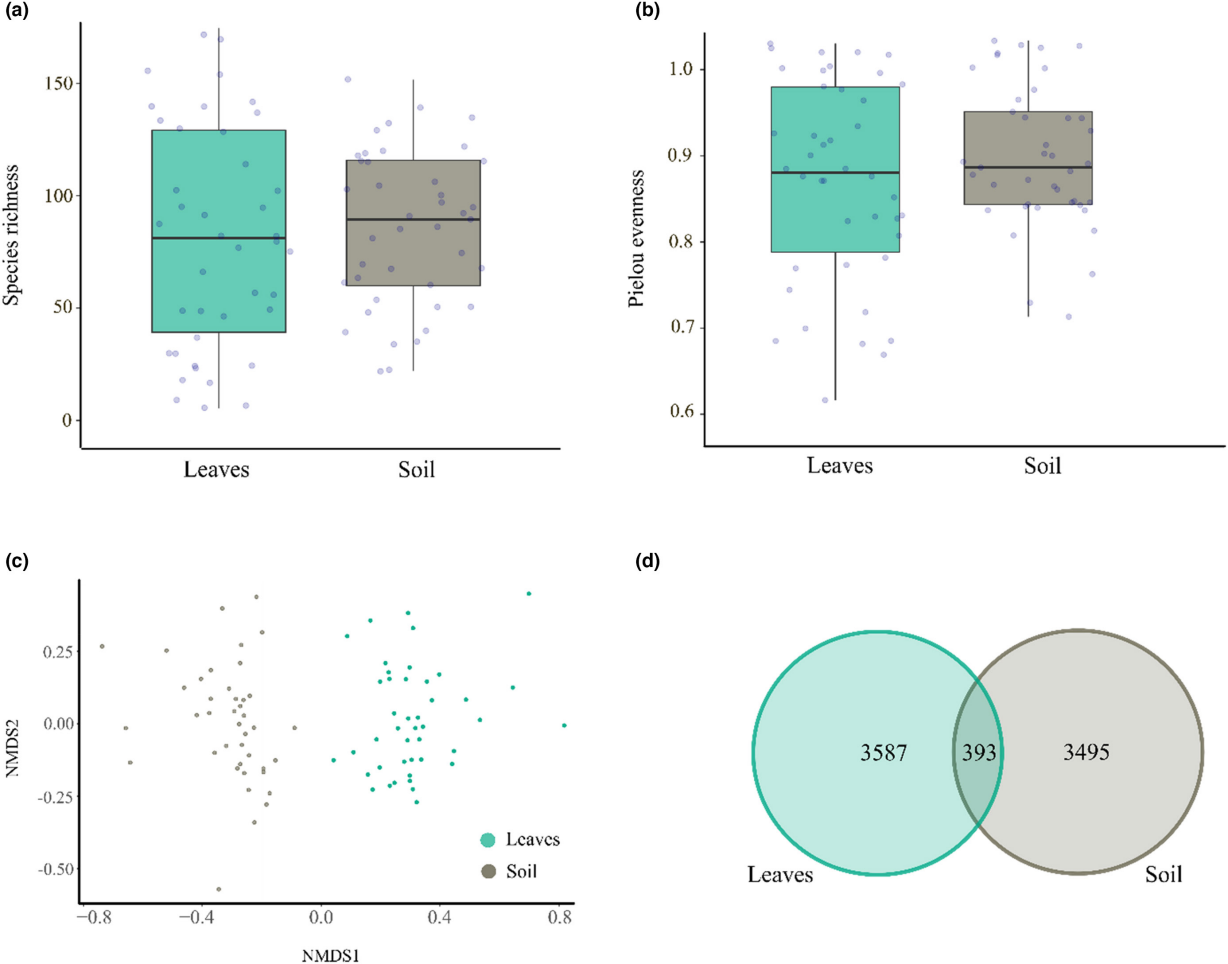
The relationship between foliar and soil fungal communities of the pedunculate oak *Quercus robur*. Box plots show (a) fungal species richness and (b) fungal evenness in the leaves and soil, respectively. In the box plots, the thick horizontal line shows the median, boxes represent the first and third quantile and whiskers represent either the minimum and maximum value or 1.5 times the interquartile range of the data. The small circles represent raw data points (total *n* = 43 for leaves and *n* = 41 for soil), which are horizontally jittered to avoid overlap. (c) Nonmetric multidimensional scaling (NMDS) of foliar and soil fungal community composition. NMDS is based on Bray–Curtis metrics of cumulative sum scaling‐normalized abundance matrices. (d) Venn diagram showing the number of amplicon sequence variants (ASVs) found exclusively in the leaves and in the soil, as well as the number of ASVs shared between them.

Species richness and evenness did not differ significantly between leaves and soil (*χ* ^2^ = 0.11, *p* = .74 and *χ* ^2^ = 1.92, *p* = .17, respectively; Figure 5a,b). Fungal community composition differed significantly between leaves and soil (*p* < .001), with only 5% of the fungal ASVs shared between leaves and soil (Figure 5c,d). We found no significant concordance in variation between the fungal community in the leaves and soil (Procrustes test: m^2^ = 0.997, *p* = .9164).

## 4 DISCUSSION

Our findings draw a contrasting picture of the spatial patterns and drivers of the fungal community associated with oak leaves and soil at the landscape scale. Overall, the fungal communities in the leaves and soil were highly distinct, and we found no statistical relationship between the fungal communities in the leaves and soil. Within trees, the foliar community was highly variable, with only a minor part of the variation found among trees. At the landscape scale, the spatial structure was stronger for the soil than for the foliar fungal community (11% and 5%, respectively), and the soil community was spatially clustered up to 50 m. Climate, tree phenology, and spatial connectivity of the host tree did not affect the distribution of the foliar and soil fungal communities at the landscape scale. Our findings thus demonstrate that soil communities exhibit a stronger spatial structure than foliar communities—a finding potentially attributable to dispersal limitation—with little or no imprint of climate, host phenology, or host distribution on the fungal community.

### 4.1 Fungal communities differ between leaves and soil

Foliar and soil fungal communities were equal in species richness and evenness, but highly dissimilar in composition, with only 5% of the total ASVs shared between the two above‐ and belowground compartments. While Beckers et al. (2016) found that poplar roots harbored more bacterial species than poplar leaves, their findings do agree with ours in the sense that they also found highly distinct below‐ and aboveground plant‐associated communities. In the current study, we did use slightly different molecular protocols to identify fungi in leaves and soil. For this reason, it is unclear whether the estimates of species richness and evenness are strictly commensurate between the two substrates. Nonetheless, samples of soil and foliage tend to come with different challenges in terms of, for example, PCR inhibitors—thus limiting the scope for identical processing of samples, at least without introducing new biases. The more interesting result here is that of no statistical association between the foliar and soil fungal communities. In other words, variations in the structure of the species composition in either microbiome community were unrelated to variation in the other, and thus one could not be used to infer the other. This absence of a statistical association between the fungal communities of foliage and soil is surprising. While we know from previous studies that soil and leaf communities differ in composition, many putative mechanisms have been proposed that link together the below‐ and aboveground plant‐associated community, such as plant‐mediated effects and movement between leaves and soil and vice versa (Abdelfattah et al., 2021; Rodriguez et al., 2009). Our findings then suggest that processes like dispersal, environmental heterogeneity, and stochasticity weaken, or wipe out, the link between the above‐ and belowground community in a natural setting.

### 4.2 Foliar communities vary strongly among leaves within trees

In terms of intra‐ versus interindividual variation in microbial communities, we found that the majority of variation in fungal species richness (70%), evenness (82%), and community composition (98%) was among leaves within a single oak, with only a minor part of the variation among oaks. The only previous study that partitioned variation in the foliar fungal community composition within and among trees found that 69% of the variation in the fungal community of the European beech (*Fagus sylvatica*) was present among leaves. The large variation observed in this study for fungi among leaves within single trees has also been found for bacteria (Laforest‐Lapointe et al., 2016; Leff et al., 2015) and insects (Gripenberg, 2007; Gripenberg & Roslin, 2005), and may thus be a general phenomenon for tree‐associated organisms. While these studies support our finding of more variation within trees than among trees, it still provides a striking quantitative contrast. In our study, only 2% of the variation in the foliar community composition could be explained by variation among trees, which is much lower than the 31% in the beech study. The high variability of foliar fungal communities within the tree canopy might be due to microclimatic differences in leaf surface temperature and humidity or leaf physical and chemical traits (Jumpponen & Jones, 2010; Mercier & Lindow, 2000). Importantly, the low variation in the foliar fungal community among trees implies that even if we detect abiotic or biotic environmental drivers of the among‐tree spatial distribution of the foliar fungal community, such drivers will only explain a very small part of the total variation in the foliar fungal community.

### 4.3 Foliar and soil communities are differently structured across the landscape

In terms of spatial structure, foliar and soil fungal communities revealed different patterns. Contrasting with the weak spatial structure detected in the foliar fungal community, soil fungal communities were spatially clustered up to 50 m. These findings suggest that fungal communities in the soil may be more strongly structured in space than fungal communities on leaves. Whether or not this is a general pattern is so far too early to judge. While several studies have focused on spatial autocorrelation in the foliar (David et al., 2016; Koide et al., 2017; Lau et al., 2013; Vaz et al., 2014; Vincent et al., 2016) or soil fungal community (Kadowaki et al., 2014; Robinson et al., 2009; Vaz et al., 2014) at regional scales, we lack direct comparisons between the spatial structure of the foliar and soil fungal communities within one and the same landscape. The only previous study that made a direct comparison found positive spatial autocorrelation of both above‐ and belowground fungal communities associated with several grass species at distances up to 50 m (David et al., 2016). Overall, we hope that similar studies, in a range of study systems, will support (or disprove) the suggestion of stronger spatial patterning of fungal communities in soil than on leaves at the landscape scale.

The stronger spatial patterning of the fungal community in the soil than in the leaves was not reflected in a stronger spatial clustering of the functional guilds in the soil. At the level of individual fungal guilds, we detected no spatial autocorrelation in the relative abundance and community composition of either foliar or soil fungi. This suggests an interesting pattern from the perspective of the oak trees: with little aggregation across the landscape, there will thus be no real hotspots of plant and soil pathogens, or stronger ectomycorrhizal associations. Contrary to these findings, previous studies have found spatial autocorrelation in the community composition of several fungal guilds, such as ectomycorrhizal fungi at distances up to few hundred kilometers (Bowman & Arnold, 2021; Matsuoka et al., 2016; Peay et al., 2007, 2012). At present, it is unclear whether these differences in patterns reported are the result of differences in host species studied, the timing of sampling within the growing season, or substrates from which fungi were described.

### 4.4 A general lack of environmental filtering

Contrary to our a priori expectation, climatic and host‐related variables did not explain spatial variation in foliar fungal species richness, evenness, community composition, or the relative abundance of functional guilds at the landscape level. This lack of environmental imprints contrasts with numerous studies which have reported a strong relationship between foliar fungal species richness and community composition, temperature, and relative humidity at the landscape but also regional scales (Campisano et al., 2017; Faticov et al., 2021; Izuno et al., 2016; Whitaker et al., 2018; Zimmerman & Vitousek, 2012). Nonetheless, the high variability of the foliar fungal community observed among leaves within a single plant individual does attest to a potential role for environmental heterogeneity in structuring foliar fungal communities at the microscale.

For soil microbial communities, we found the same lack of environmental imprints at the landscape level, and we did not detect any effect of the spatial connectivity of trees on fungal richness, evenness, community composition, or the relative abundance of functional guilds. Nonetheless, we did find spatial clustering up to a scale of 50 m. Such strong spatial autocorrelation, irrespective of environmental conditions, may suggest that soil fungal communities are more strongly limited by dispersal. Nonetheless, spatial patterning in the soil might also be driven by environmental variables unmeasured by us, by species interactions (e.g., priority effects), or by stochastic processes (Hiscox et al., 2015; Lin et al., 2015). As for any observational study, the existence of these alternative explanations points to ample scope for further work before final causation be reached.

### 4.5 Different patterns suggest different assembly processes

For foliar fungal communities, the current lack of an imprint of microclimatic conditions, host characteristics and spatial connectivity, and the weak spatial patterning across the landscape, might suggest a lack of dispersal limitation among trees at the landscape scale. Foliar fungal communities may thus conform to the “mass effects” paradigm proposed in the metacommunity framework, under which high dispersal rates can override environmental heterogeneity and homogenize fungal communities, that is, make them more similar among the trees in the landscape (Cordier et al., 2012; Whitaker et al., 2018).

For soil communities, we found a similar lack of environmental imprints, but still some spatial aggregation in terms of a higher similarity of soil fungal communities among nearby trees. These patterns are reflected in the findings from previous studies, which have explored the effect of fragmentation on soil fungi, and found an effect of connectivity on fungal composition among habitats (Grilli et al., 2017). In general, communities have been found to be more similar among more connected trees (Boeraeve et al., 2018; Vannette et al., 2016). Importantly, the extent to which the soil communities of oaks differ from those associated with other broadleaved trees in the landscape, and the extent to which the connectivity measure thus reflects an adequate representation of the landscape from a fungal perspective, is so far unknown. Based on our current findings, we may thus propose that soil communities conform to the patch‐dynamic perspective in the metacommunity paradigm but need further work to describe the landscape from a fungal perspective.

## 5 CONCLUSIONS

During recent decades, scientists have revealed the diversity of microorganisms associated with leaves and soil and realized their major contribution to plant health and ecosystem processes. Yet, we lack an understanding of how above‐ and belowground fungal communities associated with plants are distributed in space, and what processes shape this distribution. Targeting patterns at a landscape level, we found that aboveground fungal communities are highly variable among leaves within a single tree, that belowground fungal communities showed stronger spatial structure than aboveground communities, and that finer variation in the microclimate, phenology, or distribution of individual host trees did not explain spatial patterns in the above‐ and belowground community. Overall, our findings suggest that the fungal communities of the foliage and soil of host plants not only differ from each other but can be structured by different community assembly processes.

## AUTHOR CONTRIBUTIONS

**Maria Faticov:** Conceptualization (equal); data curation (lead); formal analysis (lead); investigation (lead); methodology (equal); project administration (lead); visualization (lead); writing—original draft (lead); writing—review and editing (equal). **Ahmed Abdelfattah:** Conceptualization (equal); formal analysis (supporting); funding acquisition (supporting); methodology (equal); writing—original draft (supporting); writing—review and editing (supporting). **Peter A Hambäck:** Formal analysis (supporting); supervision (supporting); writing—original draft (supporting); writing—review and editing (supporting). **Tomas Roslin:** Formal analysis (supporting); supervision (supporting); writing—original draft (supporting); writing—review and editing (supporting). **Ayco Tack:** Conceptualization (equal); formal analysis (supporting); funding acquisition (lead); methodology (equal); supervision (lead); writing—original draft (supporting); writing—review and editing (supporting).

## Supporting information


Data S1.

## Data Availability

Data associated with this manuscript have been archived in the Figshare Data Repository (10.6084/m9.figshare.22687594).
