# Supplementary material for: Different spatial structure of plant‐associated fungal communities above‐ and belowground
Source: Ecol Evol. 2023 May 21;13(5):e10065. doi: 10.1002/ece3.10065 (PMC10200691; doi:10.1002/ece3.10065)
Supplement: Supplementary file 1 — Data S1. [file ECE3-13-e10065-s001.docx]

**Supporting Information**

**Detailed description of molecular methods and bioinformatics**

To characterize the foliar and soil fungal community, we used primers targeting the internal transcribed spacer (ITS2) region (Schoch et al. 2012). We used forward primer fITS7 (Ihrmark et al., 2012) and reverse primer ITS4 (White et al. 1990), which target a 250-450 bp fragment encompassing the entire ITS2. We used a two-step PCR approach to amplify the ITS2 region from each sample. First and second PCR steps were done once for each sample. We included one negative control (i.e. wells containing nothing but PCR reagents) in each amplicon pool. In short, the first PCR reaction mixture consisted of 12.5 µL Kapa HiFi Mastermix (Kapa Biosystems, Woburn, MA, USA), 8.5 µL H_2_O, 1 µL of each primer (10 nmol/µL), and 2 µL of DNA template. Cycling conditions were 95°C for 5 min, 98°C for 1 min, 36 cycles of 98°C for 40 sec, 58°C for 40 sec, and 72°C for 15 sec, followed by a final elongation step of 72°C for 5 min. The primers for the first PCR reaction consisted of adaptor + primer, giving the forward primer 5’– ACACTCTTTCCCTACACGACGCTCTTCCGATCTGTGARTCATCGAATCTTTG – 3’ and reverse primer 5’– AGACGTGTGCTCTTCCGATCTTCCTCSSCTTATTGATATGC – 3’. In the second PCR step 15 µL PCR template, 20 µL Kapa HiFi Mastermix and 2.5 µL of each primer (10 nmol/µL) were used. The primers for the second PCR reaction consisted of Illumina handle + X8 + adaptor, with X8 denoting barcode with unique tags of 8 bp. Reaction conditions for the second PCR were as described above, but with 14 cycles instead of 25. Concentrations were measured using the Qubit dsDNA HS assay kit (Life Technologies), after which samples were pooled in equal concentrations. To test whether potential fungal DNA was amplified, we ran the PCR products on an Agilent 2100 Bioanalyzer (Agilent, Santa Clara, CA, USA).

The initial bioinformatic processing was done in the DADA2 pipeline following the standard protocol (Callahan et al. 2016). In short, we eliminated reads with quality scores less than 10 (truncQ = 10). We also performed consensus chimera removal, where chimeras were identified in each sample independently. Paired reads were assembled using the mergePairs function with a minimum overlap of 20 bp and allowing a maximum mismatch of 5% within the region of overlap. Three samples were removed due to mislabelling. When checking the quality of sequences via clustering foliar and soil fungal community composition according to the sequenced library, we detected a weak effect of libraries on foliar and soil fungal community composition. To remove this effect, we excluded 330 ASVs that were differentially abundant between the two libraries. After quality filtering and removal of sequences that appeared in negative controls, we obtained a total of 3,247,020 sequences that clustered in 12,956 ASVs for the 192 remaining samples. On average, fungal communities were represented by 16,911 reads per sample. The most abundant sequence in each ASV was used as a representative one and ASVs were identified using the UNITE database V8 (Abarenkov et al. 2010)

| **Table S1.**  Definitions of functional guilds used for fungal taxa. |  |
| --- | --- |
| 1. **Fungal guilds assigned to leaf-associated taxa** |  |
|  |  |
| **Yeasts** | Species in the groups Filobasidiales, Tremellales, Sporidiales, Microbotryomycetes, Cystobasidiomycetes, Saccharomycetales and Taphrinaceae |
| **Pathogens** | Fungal pathogens that penetrate into the leaf tissues and cause visible symptoms from the phylum Ascomycota and Basidiomycota |
| **Saprotrophs or symbiotrophs** | Fungal saprotrophs and other endophytic fungi (e.g. symbiotrophs) from the phylum Ascomycota and Basidiomycota |
| **Other** | Fungal mycoparasites, lichenized and carnivorous fungi |
| **Unknown fungi from phylum Ascomycota and Basidiomycota** | Fungi from phylum Ascomycota and Basidiomycota, which functions are unknown |
| **Unidentified fungi** | Fungi that could not be assigned to any taxa |
|  |  |
|  |  |
| 1. **Fungal guilds assigned to soil-associated taxa** |  |
|  |  |
| **Yeasts** | Species in the groups Penicillium, Trichoderma, Mortierella, Mucorales, Saccharomycetales, Tremellales, Sporidiales, and Microbotriomycetes |
| **Ectomycorrhizal fungi** | Known ectomycorrhizal species |
| **Pathogens** | Fungal pathogens from phylum Ascomycota and Basidiomycota associated with living plant roots and soil |
| **Saprotrophs and symbiotrophs** | Fungal saprotrophs and symbiotrophs (other than known ectomycorrhizal species) from the phylum Ascomycota and Basidiomycota |
| **Other** | Fungal mycoparasites, lichenized and carnivorous fungi |
| **Unknown fungi from phylum Ascomycota and Basidiomycota** | Fungi from phylum Ascomycota and Basidiomycota, which functions are unknown |
| **Unidentified fungi** | Fungi that could not be assigned to any taxa |

**Table S2.** The impact of microclimatic variables, host characteristics (autumn phenology) and tree spatial connectivity on the foliar and soil fungal richness, evenness and community composition. Shown are p-values.

|  | **Richness** | | **Evenness** | | **Community composition** | |
| --- | --- | --- | --- | --- | --- | --- |
|  | **Leaves** | **Soil** | **Leaves** | **Soil** | **Leaves** | **Soil** |
| Growing season temperature | 0.49 | 0.13 | 0.53 | 0.22 | 0.80 | 0.92 |
| Non-growing season temperature | 0.67 | 0.42 | 0.34 | 0.21 | 0.91 | 0.47 |
| Growing season relative humidity | 0.81 | - | 0.40 | - | 0.77 | - |
| Non-growing season relative humidity | 0.74 | - | 0.07 | - | 0.89 | - |
| Temperature seasonality | 0.86 | 0.08 | 0.89 | 0.21 | 0.69 | 0.61 |
| Autumn phenology | 0.57 | - | 0.52 | - | 0.51 | - |
| Tree spatial connectivity | 0.26 | 0.57 | 0.75 | 0.15 | 0.53 | 0.31 |

**Table S3.** The impact of microclimatic variables, tree autumn phenology and tree spatial connectivity on the relative abundance of fungal guilds in the leaves and soil. Shown are p-values.

|  | Yeasts | | Pathogens | | Saprotrophs and symbiotrophs | | Ectomycorrhizal fungi | | Other | | Unknown | |
| --- | --- | --- | --- | --- | --- | --- | --- | --- | --- | --- | --- | --- |
|  | **Leaves** | **Soil** | **Leaves** | **Soil** | **Leaves** | **Soil** | **Leaves** | **Soil** | **Leaves** | **Soil** | **Leaves** | **Soil** |
| Growing season temperature | 0.89 | 0.44 | 0.57 | 0.63 | 0.98 | 0.07 | - | 0.08 | 0.99 | 0.40 | 0.65 | 0.57 |
| Non-growing season temperature | 0.30 | 0.54 | 0.98 | 0.71 | 0.76 | 0.11 | - | 0.17 | 0.58 | 0.38 | 0.67 | 0.19 |
| Growing season relative humidity | 0.14 | - | 0.48 | - | 0.88 | - | - | - | 0.76 | - | 0.39 | - |
| Non-growing season relative humidity | 0.43 | - | 0.60 | - | 0.80 | - | - | - | 0.82 | - | 0.67 | - |
| Temperature seasonality | 0.23 | 0.30 | 0.56 | 0.42 | 0.62 | 0.40 | - | 0.08 | 0.60 | 0.38 | 0.44 | 0.59 |
| Autumn phenology | 0.87 | - | 0.44 | - | 0.99 | - | - | - | 0.11 | - | 0.25 | - |
| Tree spatial connectivity | 0.10 | 0.93 | 0.09 | 0.55 | 0.39 | 0.50 | - | 0.91 | 0.69 | 0.87 | 0.08 | 0.58 |

**Table S4.** Results of Mantel tests assessing spatial autocorrelation in community composition of yeasts, pathogenic fungi and ectomycorrhizal fungi. Shown are dissimilarity matrices used, explanatory variables, Mantel test statistics and P-values.

|  | Dissimilarity matrix | Explanatory variable | Mantel *r* | *P*-value |
| --- | --- | --- | --- | --- |
| Fungal guilds in the leaves |  |  |  |  |
| Yeasts | Bray-Curtis | Pairwise geographic distance | -0.09 | 0.89 |
| Pathogens | Bray-Curtis | Pairwise geographic distance | -0.03 | 0.58 |
| Fungal guilds in the soil | | | | |
| Yeasts | Bray-Curtis | Pairwise geographic distance | 0.09 | 0.12 |
| Pathogens | Bray-Curtis | Pairwise geographic distance | 0.11 | 0.13 |
| Ectomycorrhizal fungi | Bray-Curtis | Pairwise geographic distance | 0.17 | 0.07 |

**
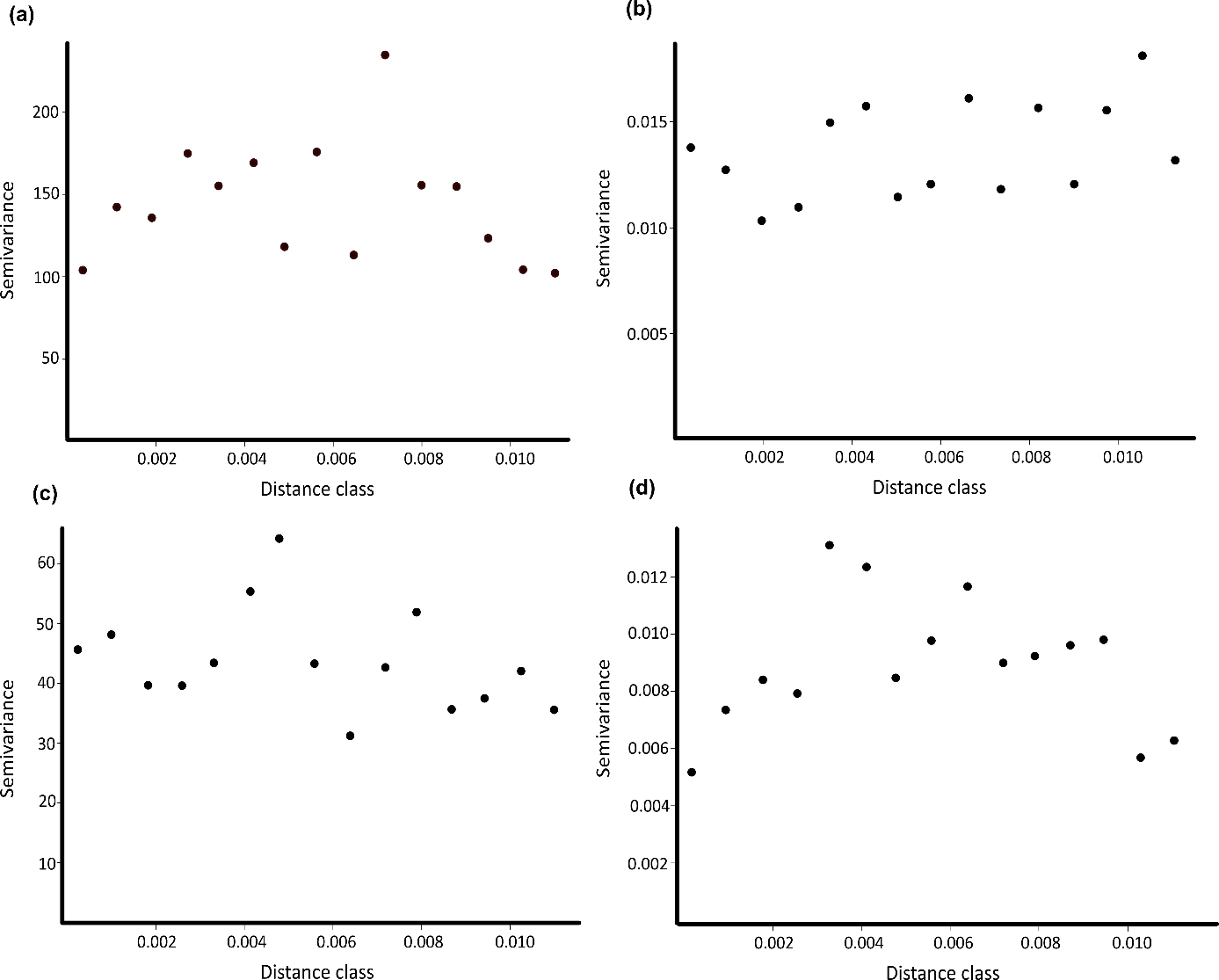
Figure S1.** Semivariograms for foliar and soil fungal community descriptors. (a) Species richness in the leaves; (b) Species evenness in the leaves; (c) Species richness in the soil and (d) Species evenness in the soil.

**Figure S2.** Semivariograms for functional guilds described from leaves. (a) Relative abundance of yeasts; (b) Relative abundance of pathogens; (c) Relative abundance of saprotrophs and symbiotrophs; (d) Relative abundance of other fungi; (e) Relative abundance of fungi with unknown functions from phylum Ascomycota and Basidiomycota; and (f) unidentified fungi.


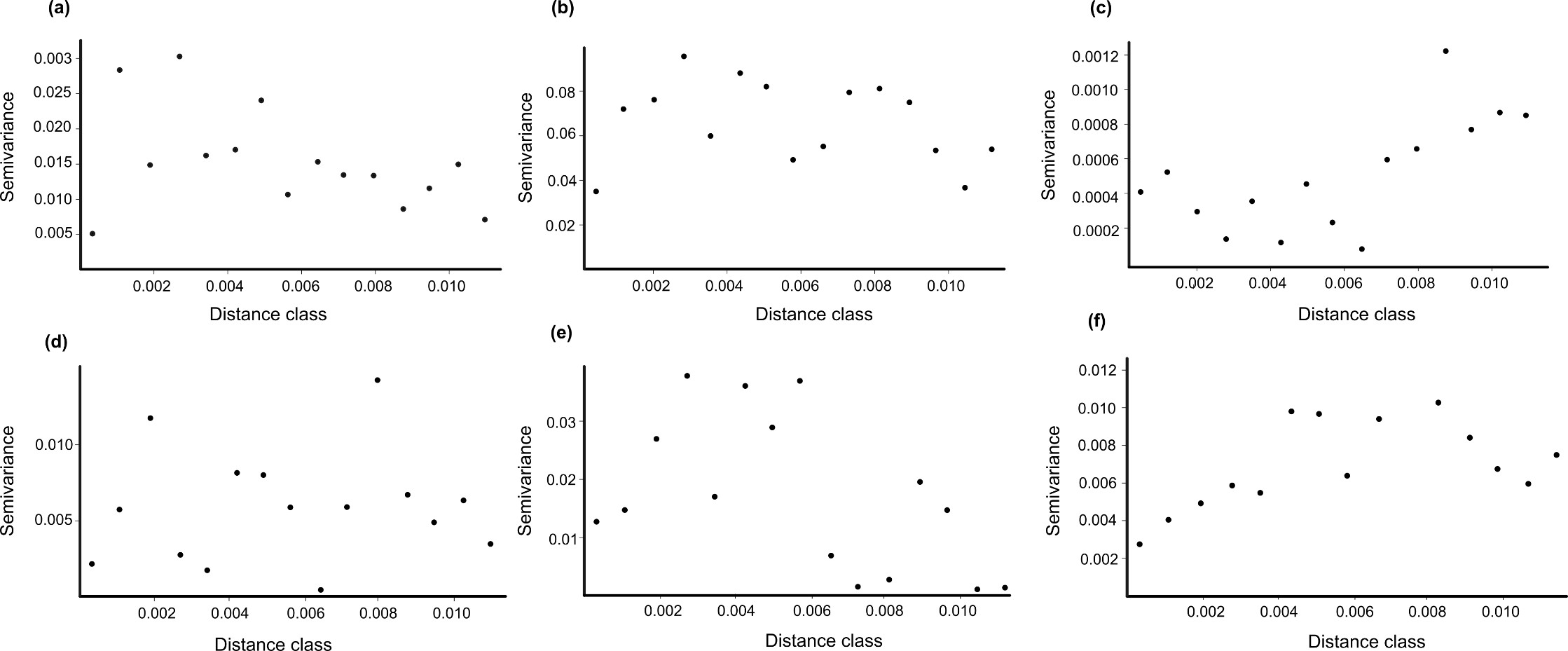


**Figure S3.** Semivariograms for functional guilds described from soil. (a) Relative abundance of yeasts; (b) Relative abundance of ectomycorrhizal fungi; (c) Relative abundance of pathogens; (d) Relative abundance of saprotrophs and symbiotrophs; (e) Relative abundance of other fungi, e.g. mycoparasites; (f) Relative abundance of fungi with unknown functions from phylum Ascomycota and Basidiomycota; and (g) unidentified fungi.

**
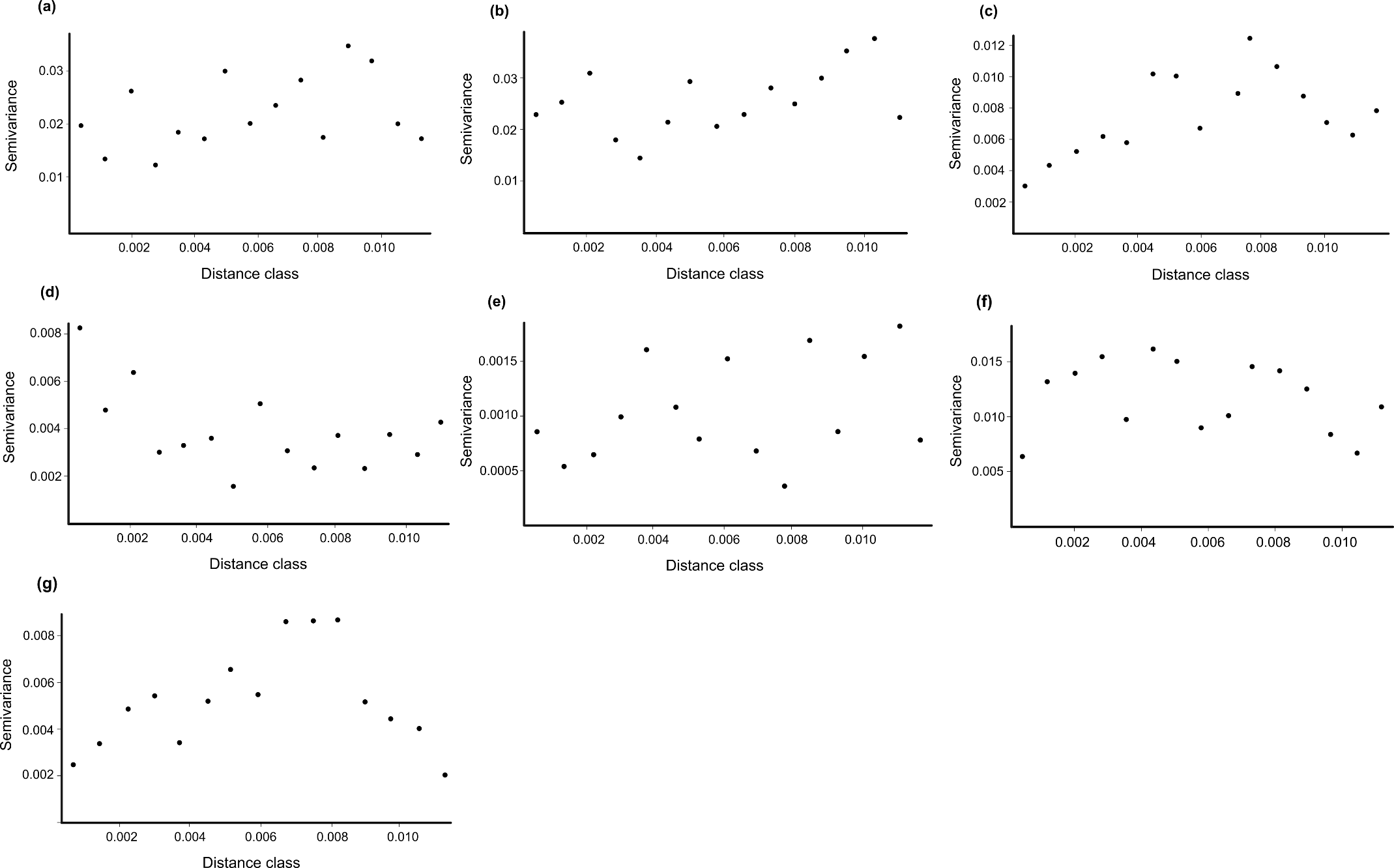
**

**Figure S4**. The relationship between microclimatic variables (temperature and relative humidity), host characteristics (autumn phenology) and tree spatial connectivity and foliar fungal richness and evenness. Panels (a) – (g) show the relationships between foliar fungal richness and growing and non-growing season temperature, growing and non-growing season relative humidity, temperature seasonality, autumn phenology and tree spatial connectivity, respectively. Panels (h) – (n) show the relationships between foliar fungal evenness and growing and non-growing season temperature, growing and non-growing season relative humidity, temperature seasonality, autumn phenology and tree spatial connectivity, respectively.


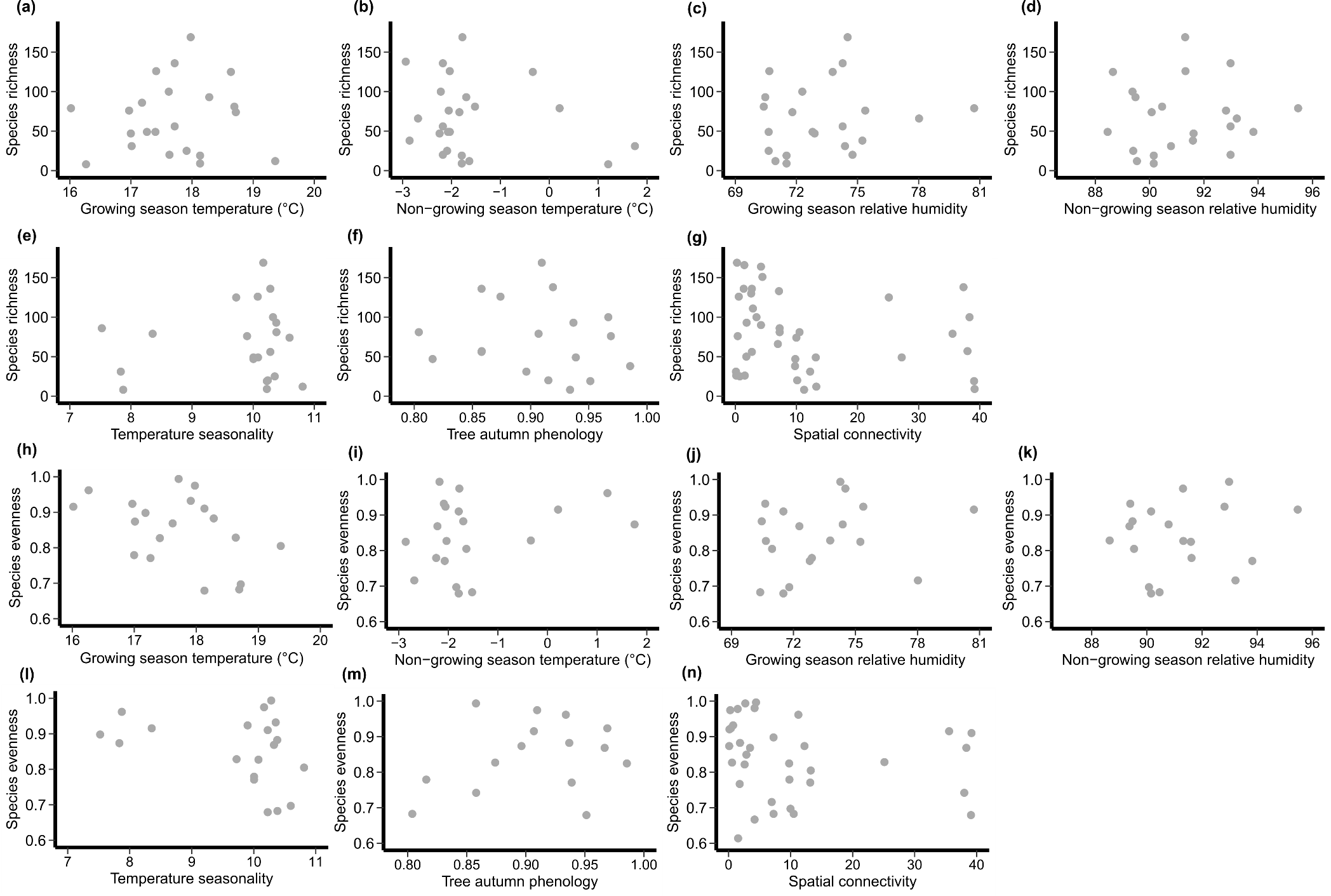


**Figure S5**. The effect of temperature and tree spatial connectivity on soil fungal richness and evenness. Panels (a) – (d) show the relationships between soil fungal richness and growing and non-growing season temperature, temperature seasonality and spatial connectivity, respectively. Panels (e) – (h) show the relationships between soil fungal evenness and growing and non-growing season temperature, temperature seasonality and tree spatial connectivity, respectively.

**
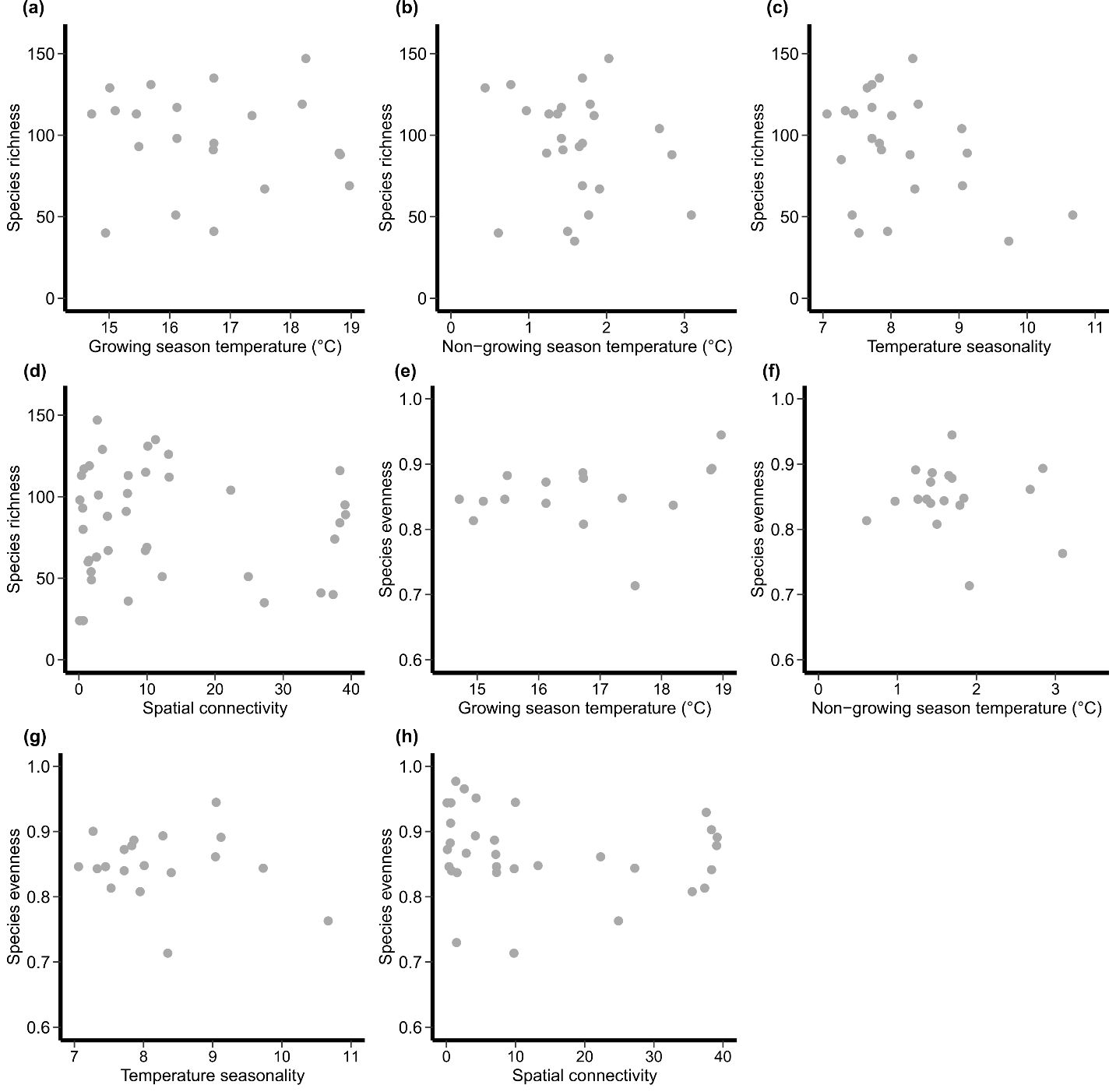
**
